# Pathways to promotion: Leveraging your network for academic success

**DOI:** 10.1002/jhm.70175

**Published:** 2025-09-30

**Authors:** Attila Nemeth, Heather Briggs, Blair P. Golden, Kierstin Kennedy

**Affiliations:** ^1^ Division of Acute Medicine VA Northeast Ohio Healthcare System Cleveland Ohio USA; ^2^ Division of Acute Medicine, Case Western Reserve University School of Medicine Cleveland Ohio USA; ^3^ Division of Hospital Medicine University of Colorado School of Medicine Aurora Colorado USA; ^4^ Division of Hospital Medicine University of Wisconsin School of Medicine and Public Health Madison Wisconsin USA; ^5^ UAB Hospital Medicine University of Alabama at Birmingham Heersink School of Medicine Birmingham Alabama USA

## INTRODUCTION

Hospitalists rise in the academic ranks at a lower rate than other specialties, with most hospitalists remaining at the instructor or assistant professor level. Literature has shown that only 11.7% of adult hospitalists have ascended to the rank of associate (9%) or full professor (2.7%).^1^, ^2^ Several factors contribute to this disparity, including substantial clinical responsibilities that limit time for research and scholarship, lack of research infrastructure, funding, and formal training, as well as the relative youth of the specialty, which can result in limited mentorship within a given institution.^1^, ^3^

Building a strong professional network is one potential strategy to fill these gaps. A professional network can facilitate your academic career development by fostering mentorship and collaboration, providing access to professional opportunities, and even protecting against burnout.^4^, ^5^, ^6^ Within hospital medicine, the ability to cultivate strong professional relationships across institutions may be particularly important given the potential scarcity of promoted hospitalist faculty at your own institution. This paper highlights the importance of networking when pursuing academic promotion, offers strategies on building a strong professional network, and provides tips for how you can support junior faculty after your successful promotion.

## WHY IS A PROFESSIONAL NETWORK IMPORTANT FOR PROMOTION?

Networking is crucial to building a robust portfolio for promotion. In fact, the reach of your network has a positive correlation with success in academic promotion.^7^ Leveraging the skill of networking to build relationships in advance can enhance knowledge, promote professional development, foster innovation, and create opportunities for collaboration and scholarship, as well as create a ready pool of potential colleagues to support your promotion candidacy, by demonstrating a reputation beyond your immediate sphere of practice. For example, collaboration with individuals outside of your organization can enhance your visibility and convey your reputation to others with whom you are not directly connected.^7^ Building a network of colleagues with similar professional interests and scholarly activity with whom you can collaborate can yield effective letters of support because these individuals can speak to the caliber and impact of your work. Peer mentors may also provide mutual support and offer a fresh perspective on challenges and opportunities available at your own institution. Strategic networking with colleagues from other institutions also allows them to speak to the transferability of your work beyond your home institution and how you measure against another institution's standards of promotion.

Furthermore, networking can help increase your ability to navigate the promotion process, which can be unfamiliar and stressful for junior faculty.^8^ Leveraging the experience of more senior faculty can not only make the process less intimidating but also offer insights on how to build a stronger portfolio and avoid common pitfalls.^9^

## STRATEGIES FOR BUILDING YOUR NETWORK

Networking is an intentional and iterative process to expand your professional connections. We offer strategies to help faculty navigate this process.

First, as an early‐career faculty, you should seek mentors and sponsors who can facilitate collaborations in scholarly activities.^10^ While a mentor, someone who lends their expertise to help guide you, can act as a sponsor, a sponsor, someone who helps advance your career through influence and advocacy, may not be suited to serve as a mentor; therefore, it is wise to seek both.^11^ A single mentor is unlikely to address every need, so you should strive to create a mentorship team, a small group of mentors with complementary expertise who can provide perspective, guidance, and accountability.^12^ Having a mentorship team within and outside of your home institution may offer balanced perspectives and promote a sense of community.^12^, ^13^ Additionally, coaches and connectors can help you strategically expand your network.^10^

Next, early‐career faculty should engage in scholarly activities. Start by reflecting on your academic interests and goals, then pursue opportunities that help you build your academic niche. Martin et al. discuss the development of a working group that promotes cross‐institutional publications such as the VA Hospital Medicine Academic Collaborative.^14^ Promotions committees look for evidence of regional or national expertise, which can be demonstrated through scholarship and visibility in the field. However, changes are on the horizon as recent publications have suggested expanding the approach of promotion committees from considering published papers to “emphasizing a scholarly approach to teaching and expanding the definition of education scholarship.”^15^

Collaborations on local and national projects serve a dual purpose. They can lead to scholarly products, such as peer‐reviewed publications or workshops at professional society meetings, and help expand your professional network. To make the most of professional meetings, you should take an active approach to building connections. Prepare by crafting and practicing an “elevator pitch” about your work and bring business cards (print or digital) to facilitate post‐meeting connections. Attend sessions to meet potential collaborators and ask senior colleagues to facilitate introductions to others who share your interests. Joining special interest groups or committees within professional societies is another effective way to meet other faculty with shared interests. Additionally, participating in smaller‐group training programs aligned with your career goals, such as the Academic Hospitalist Academy, regional SHM meetings, or programming at other relevant national meetings can foster deeper relationships that support your academic development. Be assertive about communicating your interests, career objectives, and intention to build productive collaborative relationships!

An additional step involves maximizing your social media presence.^16^ While no common community platform currently exists, thought leaders often post on Bluesky, LinkedIn, and professional society blogs and message boards. Building connections on these platforms can broaden your network and create opportunities for future collaboration. The Journal of Hospital Medicine's Innovations Corner includes a QR code to facilitate connection to authors as well.^17^ While promotions criteria have been slow to evolve, some medical schools are also increasingly recognizing digital scholarship as part of promotions considerations.^18^, ^19^ If nontraditional outlets for dissemination will be a substantial part of your scholarly portfolio, seeking advice early from your mentor, chief, and promotions committee to position yourself for career advancement is critical.

Finally, as your professional network of colleagues grows, it is vital to maintain and nurture your relationships. Continued attendance and presentations at professional meetings to maintain your visibility and build new collaborations is essential as you develop your academic portfolio. Connections could facilitate future research collaborations or even lead to new leadership opportunities.

## STEPS AFTER SUCCESSFUL PROMOTION: ASSESSING GOALS AND “PAYING IT FORWARD”

Once you have been successfully promoted to associate professor, you may need to reassess your career responsibilities and priorities. New faculty are often encouraged to say “yes” to most opportunities to explore and determine their professional niche and scholarly focus. However, this can result in commitments that no longer align with your new priorities. Maleque et al. have highlighted the importance of identifying one's values (such as leadership) and aligning these interests with the needs of their institution.^20^ They describe an approach to narrow the scope of faculty interest by conducting a needs assessment, reviewing priorities, and leveraging the network faculty have developed. In “The Secret to Saying No,” Shah provides a decision framework through which to vet new opportunities and relinquish existing commitments.^21^ Additionally, you may need to reevaluate your goals, as well as the makeup of your mentorship team, to ensure continued alignment and support as your career advances and focus evolves. Well aligned mentors and sponsors can also provide feedback on how potential new opportunities and responsibilities align with updated goals.^22^

Once the needs assessment has been completed, you can “pay it forward” as a new associate professor by identifying junior faculty to mentor within the volunteer roles that the associate professor no longer fills. For example, an early‐career hospitalist has an interest in improving the response to patients with sepsis. As a nonfunded committee member, they work on hospitalist division‐specific responses to that patient population. But over time, they expand that role to a hospital system‐wide program, and the hospitalist has advanced to the level of associate professor. Given the change in responsibility for the associate professor, a mentee can now assume the role at the division level. Once promoted associate professors can also serve as a resource for more junior faculty, helping them navigate a successful pathway to promotion and utilizing their connections to assist other faculty in expanding their own networks.

Intentionally building and nurturing your professional network is vital to successful academic advancement in hospital medicine. By fostering and maintaining connections across institutions, engaging in scholarly activities, and enhancing your social media presence, you can strengthen your network. Collaborative relationships can foster the evolution of an academic body of work, develop your professional interests, and support the development of a successful promotion portfolio. The network built through this process can also be leveraged to support junior faculty ensuring future success for the field of academic hospital medicine.

## CONFLICT OF INTEREST STATEMENT

The authors declare no conflicts of interest.

## References

[1] Sumarsono A , Keshvani N , Saleh SN , et al. Scholarly productivity and rank in academic hospital medicine. J Hosp Med. 2021;16(9):545‐548. 10.12788/jhm.3631 34197300

[2] Pappas MA , Jenkins AM , Horstman MJ , et al. State of research in adult hospital medicine: updated results of a national survey and longitudinal analysis of national data. J Hosp Med. 2023;18(6):519‐523. 10.1002/jhm.13096 37020348 PMC10247482

[3] Felde L , Burden M , Shah N , Ramos P , Chu ES . Characteristics of adult hospital medicine fellowships in the United States: a cross‐sectional survey study. J Hosp Med. 2023;18(4):287‐293. 10.1002/jhm.13052 36779314

[4] Salib S , Hudson FP . Networking in academic medicine: keeping an eye on equity. J Grad Med Educ. 2023;15(3):306‐308. 10.4300/JGME-D-22-00546.1 37363681 PMC10286928

[5] Del Carmen MG , Herman J , Rao S , et al. Trends and factors associated with physician burnout at a multispecialty academic faculty practice organization. JAMA Netw Open. 2019;2(3):e190554. 10.1001/jamanetworkopen.2019.0554 30874776 PMC6484653

[6] Herzke C , Bertram A , Stein A , Yeh H‐C , Cofrancesco Jr. J. Promotion of academic hospitalists: room for improvement. Am J Hosp Med. 2021;5(4):2021. 10.24150/ajhm/2021.016

[7] Warner ET , Carapinha R , Weber GM , Hill EV , Reede JY . Faculty promotion and attrition: the importance of coauthor network reach at an Academic Medical Center. J Gen Intern Med. 2016;31(1):60‐67. 10.1007/s11606-015-3463-7 26173540 PMC4700018

[8] Mbuagbaw L , Anderson LN , Lokker C , Thabane L . Advice for junior faculty regarding academic promotion: what not to worry about, and what to worry about. J Multidiscip Healthc. 2020;13:117‐122. 10.2147/JMDH.S240056 32099379 PMC7002385

[9] Maleque N , Thomas M , Greysen R , Khawaja H . Pathways to promotions: strategies to get started. J Hosp Med. 2026;21:207‐209. 10.1002/jhm.70178 41028959

[10] Chopra V , Arora VM , Saint S . Will you be my mentor?—Four archetypes to help mentees succeed in academic medicine. JAMA Intern Med. 2018;178(2):175‐176. 10.1001/jamainternmed.2017.6537 29181497

[11] Shah SS , Spector ND . The art of sponsorship: priming talent for success in academic medicine. J Hosp Med. 2024:1‐3. 10.1002/jhm.13517 39344304

[12] Shah SS . Beyond “who is your mentor?” J Hosp Med. 2024;19(9):763‐764. 10.1002/jhm.13435 38873768

[13] Shah SS . Career development committees: a guide for early‐ and mid‐career faculty. J Hosp Med. 2024:1‐3. 10.1002/jhm.13509 39295114

[14] Martin SK , Allen‐Dicker J , Ricotta DN , Kwan BK . The Academic Catalyst Group: a tactical framework for working groups to enhance clinician‐educator academic career development. Acad Med. 2025;100(1):7‐11. 10.1097/ACM.0000000000005835 39083625

[15] Chang A , Karani R , Dhaliwal G . Mission critical: reimagining promotion for clinician‐educators. J Gen Intern Med. 2023;38(3):789‐792. 10.1007/s11606-022-07969-5 36456843 PMC9971380

[16] How to network on social media like a pro. Business Development Bank of Canada. Accessed December 5, 2024. https://www.bdc.ca/en/articles-tools/entrepreneurial-skills/improve-networking/5-tips-to-network-on-social-media-like-a-pro

[17] Sata SS , Shah SS . Innovations corner: a new column in the Journal of Hospital Medicine. J Hosp Med. 2024;19(6):447‐448. 10.1002/jhm.13369 38647265

[18] Johng SY , Mishori R , Korostyshevskiy VR . Social media, digital scholarship, and academic promotion in US medical schools. Fam Med. 2021;53(3):215‐219. 10.22454/FamMed.2021.146684 33723821

[19] Amin M , Trubitt M , O'Glasser AY , Brooks MN . Publish or perish: a path forward for digital scholarship. J Hosp Med. 2025;25. 10.1002/jhm.70027 40097981

[20] Maleque NM , Hartigan S , Merchant N . Finding your niche as a generalist: from concept to action. J Hosp Med. 2023;18(3):270‐273. 10.1002/jhm.13034 36564957

[21] Shah SS . The secret to saying no: a decision framework for physicians. J Hosp Med. 2025:1‐4. 10.1002/jhm.13590 39821555

[22] Niazi M , Mahboob U , Shaheen N , Gul S , Saeed MHB , Kiyani A . Exploring the factors affecting career progression in informal faculty mentoring sessions within mentor and mentee relationships: a qualitative study. BMC Med Educ. 2024;24(1):1242. 10.1186/s12909-024-06170-y 39482650 PMC11529315

